# Transcriptional regulation by Ferric Uptake Regulator (Fur) in pathogenic bacteria

**DOI:** 10.3389/fcimb.2013.00059

**Published:** 2013-10-02

**Authors:** Bryan Troxell, Hosni M. Hassan

**Affiliations:** ^1^Department of Immunology and Microbiology, Indiana University School of MedicineIndianapolis, IN, USA; ^2^Prestage Department of Poultry Science, North Carolina State UniversityRaleigh, NC, USA

**Keywords:** Ferric Uptake Regulator, iron, oxidative stress, gene regulation, pathogenic bacteria

## Abstract

In the ancient anaerobic environment, ferrous iron (Fe^2+^) was one of the first metal cofactors. Oxygenation of the ancient world challenged bacteria to acquire the insoluble ferric iron (Fe^3+^) and later to defend against reactive oxygen species (ROS) generated by the Fenton chemistry. To acquire Fe^3+^, bacteria produce low-molecular weight compounds, known as siderophores, which have extremely high affinity for Fe^3+^. However, during infection the host restricts iron from pathogens by producing iron- and siderophore-chelating proteins, by exporting iron from intracellular pathogen-containing compartments, and by limiting absorption of dietary iron. Ferric Uptake Regulator (Fur) is a transcription factor which utilizes Fe^2+^ as a corepressor and represses siderophore synthesis in pathogens. Fur, directly or indirectly, controls expression of enzymes that protect against ROS damage. Thus, the challenges of iron homeostasis and defense against ROS are addressed via Fur. Although the role of Fur as a repressor is well-documented, emerging evidence demonstrates that Fur can function as an activator. Fur activation can occur through three distinct mechanisms (1) indirectly via small RNAs, (2) binding at *cis* regulatory elements that enhance recruitment of the RNA polymerase holoenzyme (RNAP), and (3) functioning as an antirepressor by removing or blocking DNA binding of a repressor of transcription. In addition, Fur homologs control defense against peroxide stress (PerR) and control uptake of other metals such as zinc (Zur) and manganese (Mur) in pathogenic bacteria. Fur family members are important for virulence within bacterial pathogens since mutants of *fur*, *perR*, or *zur* exhibit reduced virulence within numerous animal and plant models of infection. This review focuses on the breadth of Fur regulation in pathogenic bacteria.

## Introduction

Transition metals are essential elements in biological systems. Metabolic pathways, DNA synthesis, RNA synthesis, and protein synthesis are dependent on the availability of the appropriate metal cofactor. In support of this, all cells have designated gene products that transport metals to maintain cellular function; however, certain essential metals cause the formation of toxic reactive oxygen species (ROS). In the earliest description of what is now known as the Fenton reaction, iron (Fe) was shown to act catalytically in the oxidation of tartaric acid (Fenton, 1894). The Fenton reaction produces the hydroxyl radical (HO.), a ROS capable of oxidizing macromolecules and lipids (Imlay et al., 1988; Lloyd et al., 1997). Therefore, cells must tightly regulate the concentration of Fe to avoid ROS-mediated cell damage.

**Figure 1:**
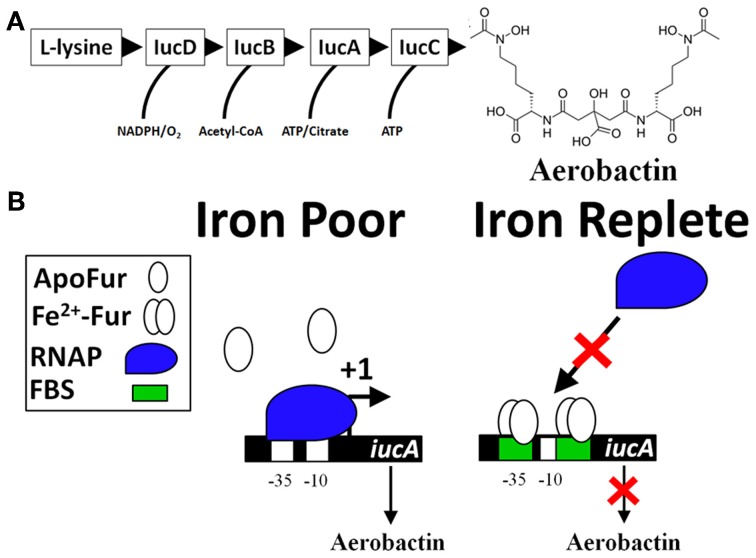
**The classic model of Fur repression of iron acquisition (*iucA* as an example). (A)** Biosynthesis of the siderophore aerobactin requires several genes located in an operon (*iucABCD, iutA*). Expression of the initial gene, *iucA*, is Fur-repressed (De Lorenzo et al., 1987) and production of aerobactin is known to be produced by virulent strains of bacteria, especially strains causing disease in avian hosts (i.e., Avian pathogenic *E. coli* or APEC) (Lafont et al., 1987; Xiong et al., 2012; Ling et al., 2013). The sequential enzymatic activity of IucD, IucB, IucC, and IucA convert L-lysine into aerobactin, a potent Fe-scavenging siderophore. **(B)** There are two Fur-binding sites (FBS) for Fe-dependent regulation of *iucA*. Both FBS are located within the P1 promoter (overlapping the −35 and also the −10 sites). Under conditions of Fe-deprivation (left panel), there is increased transcription (signified by a +1) of the *iucABCD* genes whose protein products form a biosynthetic pathway that produces aerobactin. Under Fe-replete conditions (right panel), Fur binds to DNA at the FBS (green box) and blocks access of the −35 and −10 sites by RNA polymerase (RNAP, blue shape).

Bacteria sense their environment and alter expression of genes that promote survival. This is accomplished by transcription factors that regulate expression of beneficial or detrimental genes. In order to acquire Fe in Fe-limiting environments, bacteria and fungi synthesize and secrete low molecular weight compounds, called siderophores, which have high affinity for binding Fe^3+^. Most siderophores are produced by the non-ribosomal peptide synthesis (NRPS) pathway and an example is the siderophore enterochelin. The final steps of the pathway are executed by the action of the Ent proteins (encoded by the *entD*, *entF*, and *entCEBA* genes) (Gehring et al., 1998; Salvail et al., 2010). Aerobactin, another siderophore, is sequentially produced by the proteins IucD, IucB, IucA, and IucC (Figure 1A) that are expressed in an operon (*iucABCD*). Aerobactin is an example of a siderophore not produced by the NRPS pathway. Transcriptional control of both siderophores is regulated by the concentration of intracellular Fe^2+^ (Bagg and Neilands, 1987b); when intracellular Fe^2+^ is low, the model bacterial organism, *Escherichia coli* induces siderophore production (Brot and Goodwin, 1968; Bryce and Brot, 1971). The Fe-bound siderophores are subsequently transported into the cell to satisfy an Fe^2+^ requirement. Because Fe^2+^ transcriptionally controls expression of gene products that promote iron acquisition, Fe^2+^ was predicted to be a corepressor for a DNA-binding protein. Isolation of a mutant of *Salmonella enterica* subsp. *enterica* serovar Typhimurium (*S*. Typhimurium) that constitutively expresses iron uptake proteins supported this hypothesis (Ernst et al., 1978). A mutation in Ferric Uptake Regulator (Fur) encoded by the *fur* gene was identified in *E. coli* mutants that exhibited constitutive expression of iron uptake genes (Hantke, 1981, 1984; Bagg and Neilands, 1985). Fur is a DNA-binding protein that recognizes specific DNA sequences, utilizes Fe^2+^ or Mn^2+^ as a corepressor, and blocks transcription of target genes (Bagg and Neilands, 1987a; De Lorenzo et al., 1987). Not surprisingly, the transcriptional control of *entD*, *entF*, *entCEBA*, and *iucABCD* is negatively regulated by Fur (De Lorenzo et al., 1987; Brickman et al., 1990; Stojiljkovic et al., 1994; Tsolis et al., 1995; Bjarnason et al., 2003; McHugh et al., 2003; Troxell et al., 2011a).

The collective work supports a simple model for the molecular mechanism of Fur repression that consists of Fur binding to *cis* regulatory elements of a gene and preventing the binding of the RNA polymerase holoenzyme (RNAP) (Figure 1B) (De Lorenzo et al., 1987; Escolar et al., 1999, 2000; Hantke, 2001; Lee and Helmann, 2007; Carpenter et al., 2009). As a transcriptional repressor, Fur-Fe^2+^ homodimer binds to the operator site of a target promoter (Ernst et al., 1978; Bagg and Neilands, 1985, 1987a; Neilands, 1993; Escolar et al., 1997, 1998). However, Fur can form a multimeric complex with DNA sequences extending beyond the operator site (Escolar et al., 2000; Baichoo and Helmann, 2002; Lavrrar et al., 2002). Initial studies defined the Fur-binding site (the Fur box) as an ≈19 bp DNA sequence with dyad symmetry, GATAATGATAATCATTATC (Calderwood and Mekalanos, 1987, 1988; De Lorenzo et al., 1987; Stojiljkovic et al., 1994). Insertion of this sequence into an operator site in the promoter of a non-Fe^2+^ regulated gene results in derepression under Fe^2+^-limiting conditions (Calderwood and Mekalanos, 1988). In an elegant approach to define Fur regulated genes within bacteria, a high copy number plasmid containing randomly cloned DNA sequences from Gram positive and negative bacteria were transformed into an *E. coli* strain that harbored a single copy of a *fhuF::lacZ* reporter fusion (Hantke, 1987). Fur represses transcription of the *fhuF* gene, which encodes a protein involved in the acquisition of Fe^3+^ (Hantke, 1983, 1987). If the cloned DNA fragment on the high copy number plasmid contains a Fur-binding site, then Fur proteins will be titrated away from the promoter of *fhuF* resulting in derepression of the *fhuF::lacZ* fusion, which can be qualitatively detected during growth on MacConkey agar plates or quantified by a β-galactosidase assay. This assay is called the Fur titration assay (FURTA) and has been used to study Fur regulation for nearly 20 years (Stojiljkovic et al., 1994; Tsolis et al., 1995; Baumler et al., 1996; Fassbinder et al., 2000; Osorio et al., 2004; Haraszthy et al., 2006; Jackson et al., 2010; Tanabe et al., 2010). *In toto*, these works solidified the role of Fur as a Fe^2+^-dependent transcriptional repressor. However, global gene expression studies have identified numerous genes that require Fur for expression (Foster and Hall, 1992; D'Autreaux et al., 2002; Bjarnason et al., 2003; McHugh et al., 2003; Troxell et al., 2011a).

### Multifactorial roles of Fe^2+^-Fur regulation in bacteria

**Figure 2:**
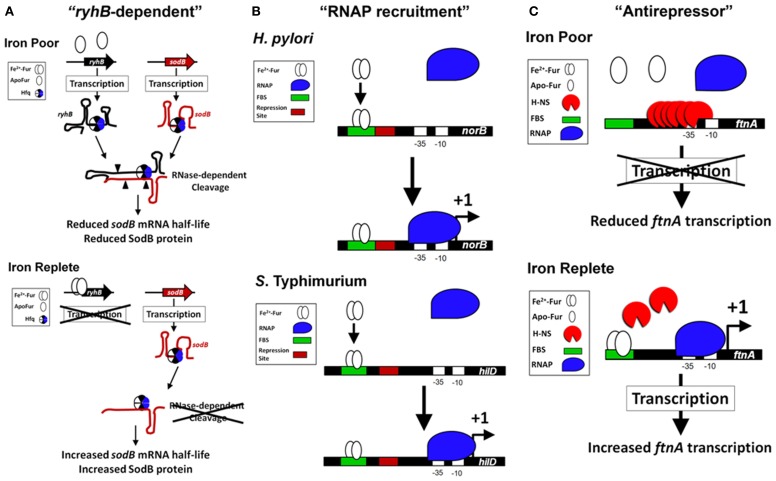
**Models of the Fur-dependent activation of gene expression in bacteria. (A)** Fur activation through “*ryhB*-dependent” mechanism (SodB as an example). Fur is indirectly required for the expression of the FeSOD (SodB) in bacteria through the sRNA *ryhB* (Masse and Gottesman, 2002; Ellermeier and Slauch, 2008). Under conditions of Fe^2+^ depletion (top panel), Fur is unable to directly repress transcription of the sRNA *ryhB* (or its paralog). This results in an increase in the level of *ryhB* within the cell. The RNA chaperone Hfq binds to *ryhB* and to the target mRNA of *sodB* (Afonyushkin et al., 2005; Urban and Vogel, 2007), which through the RNase-dependent cleavage (cleavage sites are signified by filled triangles) reduces the half-life of *sodB* mRNA and reduces SodB protein within the cell. The Fur activation of *sodB* is diminished in the absence of Hfq or *ryhB* (Masse and Gottesman, 2002; Ellermeier and Slauch, 2008; Troxell et al., 2011a). When Fur is activated during Fe^2+^ replete conditions (bottom panel), transcription of *ryhB* is blocked, which increases the half-life of *sodB* mRNA allowing for enhanced production of SodB protein and FeSOD activity. **(B)** Fur activation through “RNAP recruitment” mechanism (Examples from *S*. Typhimurium and *H. Pylori*). *In vitro* transcription assays with *H. pylori norB* regulatory sequences (Delany et al., 2004) and *S*. Typhimurium *hilD* regulatory sequences (Teixido et al., 2011) demonstrate an active Fur-Fe^2+^ binding to a FBS (signified with a green box) that promotes increased binding of the RNAP (signified with a blue shape) to the promoter and transcription of the target gene (signified with a +1). In both examples, the regulatory sequences of *norB* and *hilD* contain a repression site (signified with a red box) that may overlap the FBS (an ArsR-binding site with *norB*) or be located immediately downstream of the FBS (an H-NS binding site with *hilD*). If Fur-Fe^2+^ physically contacts the RNAP is unknown. **(C)** Fur activation through “antirepressor” mechanism (FtnA as an example). In *E. coli*, expression of the *ftnA* gene is Fur activated, but independent of the “*ryhB*-dependent” activation. Under Fe^2+^ poor conditions, H-NS binds upstream of the *ftnA* gene and represses transcription (top panel). When Fur is activated, Fur-Fe^2+^ binds to several FBS located upstream of *ftnA*, which prevents H-NS nucleation at the *ftnA* promoter and repressing transcription (bottom panel). In this example, Fur is required to block H-NS binding and can physically remove H-NS from the upstream regulatory site, which allows for *ftnA* expression.

Fur is required for the expression of several proteins within the tricarboxylic acid cycle (TCA) and the Fe^2+^-dependent superoxide dismutase (SodB) (Hantke, 1987; Gruer and Guest, 1994; Dubrac and Touati, 2000, 2002). The disruption of the TCA cycle within *fur* mutants may have a relevant role for the regulation of virulence since mutations within the TCA cycle alter virulence expression in *Staphylococcus epidermidis* and *Vibrio cholera* (Sadykov et al., 2008; Minato et al., 2013). In addition, disruption of the TCA cycle reduces *S*. Typhimurium virulence in mice (Tchawa Yimga et al., 2006; Bowden et al., 2010). The role of Fur in TCA cycle regulation is an example of how Fur regulation is multifactorial; *fur* mutants exhibit many phenotypes not just enhanced expression of siderophores. The molecular mechanism for the Fur's positive activation in the TCA cycle and SodB went unexplained until a landmark publication determined the importance of a highly conserved small untranslated RNA (sRNA) named *ryhB* in activation by Fur (Masse and Gottesman, 2002). *ryhB* is directly repressed by Fur (Vassinova and Kozyrev, 2000; Masse and Gottesman, 2002) and base pairs with target mRNAs, such as *sodB* and the succinate dehydrogenase operon *sdhCDAB*, which results in degradation of the mRNAs thereby reducing expression of the gene products (Figure 2A). Deletion of *ryhB* in a Δ*fur* results in restoration of expression of TCA proteins, SodB, and growth on succinate or fumarate minimal medium (Masse and Gottesman, 2002). Because regulation by *ryhB* requires the RNA chaperone protein, Hfq, deletion of *hfq* in Δ*fur* also restores expression of many Fur activated genes (Masse and Gottesman, 2002; Ellermeier and Slauch, 2008; Troxell et al., 2011a). *ryhB* homologs have a role in virulence, are Fur-repressed, and are encoded in the genomes of several Gram negative pathogens (i.e., *Klebsiella pneumoniae*, *Shigella*, *Vibrio cholera*, *Yersinia pestis, Salmonella*, *Pseudomonas aeruginosa*, *Neisseria meningitidis*, and *Neisseria gonorrhoeae*) (Wilderman et al., 2004; Davis et al., 2005; Mey et al., 2005a; Oglesby et al., 2005; Mellin et al., 2007; Murphy and Payne, 2007; Ellermeier and Slauch, 2008; Ducey et al., 2009; Metruccio et al., 2009; Troxell et al., 2011a; Deng et al., 2012; Huang et al., 2012; Kim and Kwon, 2013; Leclerc et al., 2013). Indirect positive regulation by Fur through negative regulation of the negative regulator, *ryhB*, is the most studied molecular mechanism for Fe^2+^-dependent activation of gene expression; however, additional evidence demonstrates that Fur may regulate virulence through more complicated mechanisms.

For example, in *S.* Typhimurium, transcription of the virulence factor *hilA* requires Fe^2+^ through Fur-dependent regulation (Thompson et al., 2006; Ellermeier and Slauch, 2008; Troxell et al., 2011b). Recently, we demonstrated enhanced transcription of *hns* in Δ*fur* and in a modified chromatin immunoprecipitation (ChIP) assay we determined that Fur bound the upstream regulatory region of *hns* in a metal-dependent manner (Troxell et al., 2011b). H-NS is known to repress transcription of *hilA* (Olekhnovich and Kadner, 2006). H-NS is a protein associated with the bacterial nucleoid and is also known as OsmZ, BglY, and PilG (Defez and De Felice, 1981; Spears et al., 1986; May et al., 1990). Deletion of *fur* and *hns* resulted in Fur-independent activation of *hilA*, which supports the indication that Fur regulation of *hilA* was indirect through H-NS (Troxell et al., 2011b). Furthermore, Fur is not required for expression of Fur-activated genes when the repressor H-NS is absent (Nandal et al., 2010; Troxell et al., 2011b) and Fur and H-NS appeared to recognize similar DNA sequences throughout the bacterial chromosome (Prajapat and Saini, 2012). In another example of the multifactorial role of Fur in bacteria, a recent study shows that Fur represses transcription of the *vvhA* gene, which encodes the major haemolysin of *Vibrio vulnificus*, yet haemolytic activity and VvhA protein level were reduced in Δ*fur* (Lee et al., 2013). Two metal-dependent proteases are responsible for degradation of VvhA, VvpE, and VvpM and transcription of *vvpE* is under negative regulation by Fur. Through genetic and biochemical approaches, it was shown that VvpE and VvpM exhibited enhanced activity in Δ*fur* resulting in reduction of the VvhA protein (Lee et al., 2013). Clearly, it can be appreciated from these two examples that the influence of Fur within the cell is global and typically involves multiple layers of regulation. Nevertheless, recent evidence indicates Fur may have a more direct role for activation of gene expression in bacteria (Figure 2).

### Mechanisms of activation of gene expression via DNA binding by fur: location, location, location

Global gene expression studies have identified genes that require Fur for expression (Foster and Hall, 1992; D'Autreaux et al., 2002; Bjarnason et al., 2003; McHugh et al., 2003; Troxell et al., 2011a). Earlier work demonstrated a unique mechanism for Fur activation in *N. meningitidis* that involves Fur directly binding to *cis* regulatory elements upstream of a Fur-activated gene (Delany et al., 2004). Unlike Fur-repressed genes that possess a characteristic Fur-binding site overlapping the RNAP-binding site, Fur-activated genes [*norB*, *pan1* (*aniA*), and *nuoA*] contain Fur boxes located ≈100 bp upstream of the transcriptional start site, while the Fur-repressed *tbp* contains a Fur box that overlaps with the RNAP-binding site. The Fur box and activation of *norB*, which encodes a protein responsible for protection against NO (Anjum et al., 2002), is conserved in *N. gonorrhoeae* (Isabella et al., 2008). Moreover, in *Helicobacter pylori*, Fur activates expression of *oorB*, which encodes a 2-oxoglutarate:acceptor oxidoreductase (Hughes et al., 1998), by directly binding to a *cis* regulatory elements located 130 bp upstream of the transcriptional start site (Gilbreath et al., 2012). The importance of OorB in virulence is demonstrated by the significant reduction in colonization of the chicken gut by a Δ*oorB* mutant strain of *Campylobacter jejuni* (Weerakoon et al., 2009). In *V. cholera*, Fur activates expression of the outer membrane porin, *ompT*, through binding a Fur box located 90 bp upstream of the transcriptional start site (Craig et al., 2011). In *S*. Typhimurium, transcription of the virulence factor *hilD* is activated by Fur through a Fur box located nearly 200 bp upstream of the transcriptional start site (Teixido et al., 2011). HilD is an AraC/XylS-type DNA-binding protein that regulates transcription of important virulence factors within *S*. Typhimurium and is required for infection (Ellermeier et al., 2005). Importantly, the sequence of the Fur box site for activated genes is virtually identical to the Fur box of repressed genes. Collectively, the molecular evidence suggests the location of the Fur box in proximity to the RNAP-binding site determines the ability of Fur to activate gene expression.

How does Fur activate gene expression? *In vitro* transcription experiments demonstrate that Fur can activate transcription of a target gene even though the Fur boxes are located ≈100 and 200 bp upstream of the transcriptional start site, respectively (Delany et al., 2004; Teixido et al., 2011). This example of Fur activation is rare, but may involve enhanced recruitment of RNAP to the promoter of target genes (“RNAP recruitment” activation model, Figure 2B). Surprisingly, addition of the Fur protein to the *in vitro* transcription assay stimulated the production of *hilD* mRNA, which suggests improved recruitment of RNAP to the promoter of *hilD* even though the Fur box is nearly 200 bp upstream of the transcriptional start site (Teixido et al., 2011). While deletion of *fur* reduces transcription of *hilD* (Teixido et al., 2011) overexpression of Fur results in little increased activation of the *hilD* promoter contrary to overexpression of a direct activator HilC, which increases *hilD*'s promoter activity by ≈5-fold (Ellermeier and Slauch, 2008). These results indicate the role of Fur in direct transcriptional activation of a target gene is complex.

Transcriptional activators that bind upstream of the RNAP-binding site have been shown to interact with the C-terminal domain of the α subunit (α-CTD) of RNAP, which promotes transcription of the target gene (Ishihama, 1992; Busby and Ebright, 1994; Ebright and Busby, 1995; Murakami et al., 1997; Hochschild and Dove, 1998). Contact between activators and α-CTD is inhibited when the upstream activator binding site is ≥100 bp upstream of the transcriptional start site (Murakami et al., 1997). Thus, transcription factor binding sites located further than 100 bp upstream of the transcriptional start site are unlikely to interact physically with the α-CTD of RNAP. However, oligomerization of the Fur protein at Fur boxes is known to occur (De Lorenzo et al., 1987; Tardat and Touati, 1993; Escolar et al., 2000; Nandal et al., 2010; Teixido et al., 2011), which suggests Fur proteins may extend to interact with other proteins nearby. Whether Fur contacts the RNAP is not known, but emerging *in vivo* evidence indicates there is another plausible molecular mechanism for Fur-dependent activation through binding DNA at a distal regulatory site.

#### Roles of Fur and H-NS in the regulation of FtnA

Fe^2+^ activates expression of the Fe-storage gene *ftnA* in a Fur-dependent manner (Masse and Gottesman, 2002; Velayudhan et al., 2007). Overexpression of *ryhB* results in the down regulation of many Fe-cofactored proteins (i.e., SodB) and increases the intracellular Fe^2+^ concentration resulting in enhanced Fur activation (Masse et al., 2005; Jacques et al., 2006). This is known as the “iron-sparing” response (Gaballa et al., 2008). Masse et al. theorized that Fur may negatively regulate a negative regulator of *ftnA*, which would manifest as a Fur activation. Evidence to support this theory was demonstrated by work from Simon C. Andrews' lab, which showed that Fur binds to a distal regulator site upstream of the RNAP-binding site in the promoter of *ftnA* to physically remove the histone-like protein, H-NS, which mediates repression of *ftnA* (Nandal et al., 2010). Unlike the activation of *norB* and *hilD*, Fur was not required for transcription of *ftnA* using *in vitro* transcription assays (Nandal et al., 2010). H-NS repressed transcription of *ftnA* and Fur was only required to relieve this repression. The role of Fur as an antirepressor in the activation of *ftnA* is supported with *in vivo* evidence: (1) *fur* is not required for *ftnA* expression in the absence of *hns*; and (2) *ftnA* expression is not reduced by Fe^2+^-chelation in Δ*hns* (Nandal et al., 2010). Fur activation of gene expression by this mode represents a 3^rd^ type of activation, the “antirepressor” activation model (Figure 2C). *In vivo* evidence supports the antirepressor model as a major mechanism for Fur-dependent activation of gene expression. Evidence for the antirepressor model is evident in *N. gonorrhoeae* because the Fur-binding site upstream of *norB* is not required for activation of expression when the *norB* repressor, ArsR, is deleted (Isabella et al., 2008). Thus, Fur antirepressor activity is an emerging model of Fur activation through DNA binding.

### Fur controls defenses against ROS

During bacterial infection the host responds to non-self molecules and initiates a potent antimicrobial response. However, bacterial pathogens are well-adapted to defending against the host antimicrobial response. In many bacterial pathogens the defense against ROS requires the Fur protein. Enzymatic defense against ROS occurs by the rapid enzymatic dismutation of superoxide (O_2−_) by superoxide dismutases (SODs) and detoxification of H_2_O_2_ by hydroperoxidases [i.e., the heme containing peroxidase/catalase (HPI), and the heme containing catalase (HPII)]. Unlike most pathogenic bacteria, *S*. Typhimurium contains 6 genes whose gene products are devoted toward degradation of H_2_O_2_. HPI (encoded by *katG*), HPII (encoded by *katE*), a Mn-dependent catalase (encoded by *katN*), an NADH-dependent alkyl peroxidase system (encoded by *ahpCF*), and two thiol specific peroxidases (encoded by *tsaA* and *tpx*). HPII and KatN are under positive regulation by the alternative σ factor RpoS, whereas HPI is induced by the redox sensing regulator OxyR during hydrogen peroxide stress (Tartaglia et al., 1989; Ivanova et al., 1994; Robbe-Saule et al., 2001; Vazquez-Torres, 2012). In addition, OxyR activates expression of *ahpC* (Storz et al., 1989; Tartaglia et al., 1989) and also *fur* (Zheng et al., 1999; Varghese et al., 2007). Regulation of *tsaA* appears Fur-independent (Delany et al., 2001) and there is a lack of evidence for whether Fe^2+^ and perhaps Fur regulate *tpx*. Deletion of any single gene or in combinations does not influence virulence; only the combined deletion of 5 out of the 6 genes results in reduced virulence signifying the importance of redundant H_2_O_2_ scavengers to virulence (Hebrard et al., 2009; Horst et al., 2010). As evident from studies in other bacterial pathogens, there are profound redundancies that contribute to resistance to H_2_O_2_ and virulence *in vivo* (Cosgrove et al., 2007; Lindgren et al., 2007; Soler-Garcia and Jerse, 2007). Because SODs and H_2_O_2_-degrading enzymes require certain metals as cofactors for enzymatic function and because Fur is a redox sensing protein (Fleischhacker and Kiley, 2011), it is not surprising that Fur is involved in the regulation of defenses against ROS.

SODs and HPI/HPII require the appropriate cofactors; Fe^2+^ is required for FeSOD (SodB) and Mn^2+^ for MnSOD (SodA) whereas heme is required for HPI and HPII function (Keele et al., 1970; Yost and Fridovich, 1973; Hassan and Fridovich, 1978; Claiborne and Fridovich, 1979; Claiborne et al., 1979). Fur directly represses transcription of the gene encoding the MnSOD (*sodA*) and indirectly activates expression of the gene encoding the FeSOD (*sodB*; Niederhoffer et al., 1990; Tardat and Touati, 1991; Beaumont and Hassan, 1993). This indirect control of *sodB* requires the RNA chaperone Hfq or *ryhB* (Masse and Gottesman, 2002; Ellermeier and Slauch, 2008; Troxell et al., 2011a). In addition, Fur controls HPI/HPII activity in a complex manner that may depend on the ability of Fur to regulate biosynthesis of the heme cofactor (Hamza et al., 2000; Benov and Sequeira, 2003; Hoerter et al., 2005; Mey et al., 2005a; Gaballa et al., 2008) (R. Saah and H. M. Hassan, unpublished data). Surprisingly, despite the enhanced transcription of *sodA* in Δ*fur*, a corresponding increase in MnSOD activity was not observed due to the increased [Fe^2+^] in the mutant. Indeed, increase in MnSOD activity in Δ*fur* was only discernible upon supplementation of the growth medium with excess [Mn^2+^] in order to outcompete the available Fe^2+^ for the active site of MnSOD (Hassan and Schrum, 1994; Schrum and Hassan, 1994; Troxell et al., 2011a). Thus, with respect to O^−^_2_ defense Δ*fur* behaves phenotypically like Δ*sodA*Δ*sodB* under Fe^2+^ replete conditions. The Fur regulation of Mn^2+^ transport is well-documented (Patzer and Hantke, 2001; Kehres et al., 2002; Guedon et al., 2003; Ikeda et al., 2005; Runyen-Janecky et al., 2006; Perry et al., 2012). Furthermore, because *katN* encodes a Mn-containing catalase and is activated by RpoS (Robbe-Saule et al., 2001) and repressed by H-NS (Beraud et al., 2010), it is likely that Fur is involved in *katN* expression in *S.* Typhimurium. Thus, the modulation of the intracellular Mn^2+^ concentration will undoubtedly influence protection against ROS and likely virulence. In support of this, numerous studies have demonstrated the importance of Mn^2+^ in the regulation of virulence and infectivity (Boyer et al., 2002; Corbin et al., 2008; Anderson et al., 2009; Ouyang et al., 2009; Ogunniyi et al., 2010; Wu et al., 2010; Champion et al., 2011; Kehl-Fie et al., 2011; Damo et al., 2013; Troxell et al., 2013). Likewise, additional members of the Fur family of metal-dependent transcription factors either bind Mn^2+^ directly and/or regulate Mn^2+^ transport.

### Fe^2+^ sequestration by the host

Because Fur requires Fe^2+^ as a corepressor the availability of this metal controls Fur activity. Moreover, the Fe^2+^-Fur complex is inactivated by ROS and reactive nitrogen species (RNS) (D'Autreaux et al., 2002; Varghese et al., 2007), both of which are generated by the host during infection. Humans and other higher eukaryotes produce numerous proteins that sequester free Fe^2+^ and heme to deprive the pathogens of iron and meanwhile prevent the toxic formation of ROS. A potent antimicrobial response, including ROS production, produced by innate cells of the host's immune system is activated in response to detection of pathogen-associated molecular patterns (PAMPs) during bacterial infection. Innate cell activation by PAMPs initiates the synthesis of large amounts of Fe^2+^ sequestering proteins to limit the available Fe pool for the pathogen, known as “nutritional immunity” (Kehl-Fie and Skaar, 2010; Hood and Skaar, 2012) and activates signaling pathways that causes the host to reduce dietary absorption of Fe that is known as “the anemia of inflammation.” In addition, the host responds to infection by increasing the body temperature (the febrile response) as a means to inhibit bacterial growth. The antimicrobial host factors produced during activation of nutritional immunity can be inhibited by the addition of Fe (Weinberg, 1974). Furthermore, the febrile response to bacterial pathogens is antimicrobial, in part, due to the reduced ability of bacteria to acquire Fe^2+^ at febrile temperatures (Kluger and Rothenburg, 1979).

Anemia of inflammation by the host in response to infection has been known for more than 60 years (Cartwright et al., 1946; Greenberg et al., 1947; Wintrobe et al., 1947) and the host protein, hepcidin, controls this response (Nicolas et al., 2002; Nemeth et al., 2004a,b). In addition, hepcidin is a host factor that strongly reduces the absorption of dietary Fe (Shayeghi et al., 2005; Drakesmith and Prentice, 2012; Prentice et al., 2012). Because Fe^2+^ is required for cellular function within nearly all cells, limiting the availability of Fe^2+^ starves pathogens for Fe^2+^ and weakens the pathogens' ability to combat antimicrobial responses by the host. Not surprisingly, there is fierce competition for accessibility of Fe^2+^ during infection. Phagocytosis of the intracellular pathogen *S.* Typhimurium by macrophages enhances expression of the Fe^2+^ export protein ferroportin, which limits the available Fe^2+^ during intracellular residence of *S*. Typhimurium (Nairz et al., 2007). Expression of ferroportin correlates directly with reduced bacterial burden of several intracellular pathogens (Paradkar et al., 2008). Thus, the host responds to infection by sequestering Fe^2+^ from the local environment of pathogens, limits the absorption of dietary Fe resulting in a very Fe^2+^ limiting host environment, and restricts available Fe^2+^ within the phagosome.

An important host factor that controls bacterial infection is the natural resistance-associated macrophage protein 1 (NRAMP1, also known as *SLC11A1*) and several research groups determined the contribution of the *SLC11A1* locus to severity of infection within animal models (Plant and Glynn, 1976; Bradley, 1977; Skamene et al., 1982; Brown et al., 2013). *S*. Typhimurium lacking *fur* are avirulent within mice with a functional NRAMP1, whereas the isogenic parent is fully virulent. Mice lacking a functional NRAMP1 are partially resistant to infection with Δ*fur* demonstrating that Fur function is important for virulence, in part, independent of the host NRAMP1 function (Troxell et al., 2011b). Evidence indicates that Fur is functional within an unstimulated macrophage cell-line expressing either a functional or mutated NRAMP1 (Taylor et al., 2009). The NRAMP1 protein is a highly conserved transporter of divalent cations and is expressed within phagocytic cells (Cellier et al., 1995; Canonne-Hergaux et al., 2002; Cellier, 2012); NRAMP1 functions as a transporter of manganese (Mn^2+^), Fe^2+^, or cobalt (Co) and is important for acidification of the phagosome (Hackam et al., 1998; Jabado et al., 2000; Forbes and Gros, 2003). Furthermore, NRAMP1 promotes additional host factors of the antimicrobial response including production of nitric oxide (NO) (Fritsche et al., 2003, 2008; Nairz et al., 2009) and production of lipocalin-2 (also called siderocalin), which binds to bacterial siderophores thereby sequestering bacterial Fe^2+^ acquisition proteins (Fritsche et al., 2012). However, bacteria have evolved a counter defense mechanism by producing salmochelins, which are structurally distinct from enterochelin and therefore not susceptible to binding by lipocalin-2 (Smith, 2007). RNS and NO perturb Fur-Fe^2+^ function within pathogens (Mukhopadhyay et al., 2004; Richardson et al., 2006; Bourret et al., 2008). NO is a crucial factor in the antimicrobial response and its production is regulated by Fe^2+^ (Weiss et al., 1994; Melillo et al., 1997; Dlaska and Weiss, 1999). Consequently, the inability to generate NO increases the Fe^2+^ content within macrophages, splenic cells, and hepatocytes thereby increasing disease severity in animal models of infection (Nairz et al., 2013). This signifies the importance of NRAMP1 in the ability to sequester Fe^2+^ from pathogens and in general antimicrobial response.

### Control of virulence by the fur family of transcriptional regulators

The Fur protein contributes to virulence in animal models for numerous bacterial pathogens (Table 1). Although the precise mechanism for the observed attenuation of *fur* mutants is not clear, evidence indicates that a reduction in the activity of enzymes required for protection against ROS may be involved. Furthermore, virulence factors within the *fur* mutants exhibit altered expression or activity, which may additionally contribute to a decrease in virulence. Because Fur also controls expression or activity of enzymes within the TCA cycle, *fur* mutants are defective in the utilization of several carbon sources (i.e., succinate, etc.), which may contribute to the inability of *fur* mutants to cause disease within animal hosts.

**Table 1:** **Animal models of infection that require Fur for virulence**.

**Species**	**Animal host**	**References**
*Actinobacillus pleuropneumoniae*	Swine	Sheehan et al., 2003; Jacobsen et al., 2005
*Aeromonas salmonicida*	Fish	Ebanks et al., 2013
*Campylobacter jejuni*	Avian	Palyada et al., 2004
*Edwardsiella ictaluri*	Fish	Santander et al., 2012
*Haemophilus influenza*	Chinchilla	Harrison et al., 2013
*Helicobacter pylori*	Murine	Bury-Mone et al., 2004
*Helicobacter pylori*	Gerbil	Gancz et al., 2006
*Listeria monocytogenes*	Murine	Rea et al., 2004
*Pseudomonas fluorescens*	Fish	Wang et al., 2009
*Salmonella enterica* serovar Typhimurium	Murine	Velayudhan et al., 2007; Curtiss et al., 2009; Troxell et al., 2011b
*Salmonella enterica* serovar Typhi	Human macrophages	Leclerc et al., 2013
*Staphylococcus aureus*	Murine	Horsburgh et al., 2001b; Torres et al., 2010
*Vibrio cholera*	Murine	Mey et al., 2005b

There are additional transcription factors within the Fur family that require alternative metals to control gene regulation and virulence. First discovered by work in *B. subtilis* within the lab of John Helmann (Bsat et al., 1998; Mongkolsuk and Helmann, 2002), PerR is widespread in other bacteria and contributes to virulence within pathogens (Van Vliet et al., 1999; Horsburgh et al., 2001a; Rea et al., 2004, 2005; Gryllos et al., 2008). The DNA-binding activity of PerR is sensitive to relevant concentrations of H_2_O_2_ and upon metal-dependent oxidation results in derepression of target genes (Lee and Helmann, 2006). PerR homodimers are detected as two forms, one which contains two ions of Zn^2+^/Fe^2+^ per monomer and one which contains two ions of Zn^2+^/Mn^2+^ per monomer. Only the Zn/Fe form is sensitive to H_2_O_2_-induced derepression and, as expected, PerR regulates genes whose protein products detoxify H_2_O_2_ (Herbig and Helmann, 2001; Lee and Helmann, 2006). Thus, the H_2_O_2_-sensing of PerR is directly influenced by the Mn^2+^:Fe^2+^ ratio within the cell. Maintenance of the Mn^2+^:Fe^2+^ ratio is an important aspect within bacterial pathogens (Veyrier et al., 2011).

Zinc (Zn^2+^) uptake regulator (Zur) is a Fur family regulator that responds to Zn^2+^ and was discovered by two groups working with *E. coli* and *Bacillus subtilis* (*B. subtilis*) (Gaballa and Helmann, 1998; Patzer and Hantke, 1998). As expected for a Fur homolog, Zur represses transcription of Zn^2+^ uptake when bound to the corepressor Zn^2+^ (Patzer and Hantke, 2000; Gaballa and Helmann, 2002). Because ribosomal proteins utilize Zn^2+^ for activity Zur also represses transcription of genes involved in mobilization of Zn^2+^ by ribosomal protein paralogs, which may allow for protein synthesis under conditions of Zn^2+^ limitation known as the “failsafe” model (Maciag et al., 2007; Natori et al., 2007; Gabriel and Helmann, 2009). The Zur protein or Zn^2+^ uptake systems have an important role for bacterial pathogens, which demonstrate the importance of Zn^2+^ acquisition during infection (Campoy et al., 2002; Ammendola et al., 2007; Sabri et al., 2009; Smith et al., 2009; Desrosiers et al., 2010; Pesciaroli et al., 2011; Corbett et al., 2012; Dowd et al., 2012; Gielda and Dirita, 2012). The ability to acquire Zn^2+^ by bacterial pathogens is likely a broad requirement among bacterial pathogens during infection. More recently, a Fur-homolog was characterized as a Mn^2+^-dependent DNA-binding protein (Mur). This regulator, originally isolated from *Rhizobium leguminosarum* (Diaz-Mireles et al., 2004, 2005; Bellini and Hemmings, 2006), utilizes Mn^2+^ as a corepressor. In contrast to Fur and Zur, the role of Mur in bacterial pathogenesis is less understood. However, genes regulated by Mur are important for virulence in the pathogen *Brucella abortus* (Anderson et al., 2009; Menscher et al., 2012), which indicates Mur function may be important to virulence.

## Conclusions

The Fur family of transcriptional regulators control virulence, defense against ROS, and transport of Fe^2+^, Zn^2+^, and Mn^2+^. Because of the anemia of inflammation and nutritional immunity exerted by the host during infection, metals are in low abundance in response to infection. In this metal-poor environment, the demetaleted Fur would allow for efficient acquisition of iron and enhances the fitness of the pathogen; however, deletion of *fur* most often results in partial or complete attenuation within animal models of infection. Notably, deletion of *fur* results in reduced expression of active enzymes responsible for defense against ROS, reduced expression of key metabolic pathways, and reduced expression of important virulence factors. This signifies that Fur's critical contribution to virulence may not be due to its classical role as a transcriptional repressor of metal acquisition (Figure 1), but to its complex role as a transcriptional activator of virulence (Figure 2).

### Conflict of interest statement

The authors declare that the research was conducted in the absence of any commercial or financial relationships that could be construed as a potential conflict of interest.
